# Nutritional enhancement in black seed (*Nigella sativa* L.) using bacteria‐based biofertilizers

**DOI:** 10.1002/fsn3.3982

**Published:** 2024-03-18

**Authors:** Nayyab Naeem, Arusa Aftab, Humaira Rizwana, Zill‐e‐Huma Aftab, Zubaida Yousaf, Zainab Maqbool, Zainab Shahzadi

**Affiliations:** ^1^ Department of Botany Lahore College for Women University Lahore Punjab Pakistan; ^2^ Department of Botany and Microbiology King Saud University Riyadh Riyadh Saudi Arabia; ^3^ Department of Plant Pathology University of the Punjab Lahore Punjab Pakistan

**Keywords:** antioxidant assay, *Azospirillum lipoferum*, *Nigella sativa*, *Pantoea agglomerans*, phytochemicals

## Abstract

*Nigella sativa* L. is an aromatic spice, utilized as an original and peculiar flavoring ingredient in a variety of culinary applications and pharmaceuticals. Black seed (*Nigella sativa* L.) belongs to the family Ranunculaceae. It is an undercultivated crop in Pakistan. The present study was planned keeping in mind sustainable development goals SDG 3 (good health and well‐being) and SDG 15 (life on land). The effects of several rhizospheric bacterial strains and synthetic fertilizers on the development of *N. sativa* and nutrition were studied using a completely randomized experimental design. For this purpose, plant growth‐promoting effects of different strains (*Azospirillum brasilense, Azospirillum lipoferum*, and *Pantoea agglomerans)* and synthetic fertilizers (nitrogen and phosphorus) were assembled to check their effects individually and in combination form*. Azospirillum lipoferum* and *Pantoea agglomerans* inoculation significantly enhanced the morphological characteristics of *N. sativa*, whether applied individually or in combination, with positive effects on seedlings, plant height, number of branches, number of leaves, number of flowers, stamens numbers, follicles number, number of tentacles and seed production. *N. sativa* plants that were simultaneously inoculated with *Azospirillum lipoferum* and *Pantoea agglomerans* showed the highest potential for antioxidant activity, particularly in petroleum ether extracts. In the methanolic extract, a higher amount of radical scavenging was observed as compared to positive and negative control. There was also increase in fat, moisture and carbohydrate contents of the combination inoculated plant. So, from the present study, in Pakistan, the technique is recommended to enhance the yield and nutritional value of *N. sativa*.

## Graphical abstract

Nigella sativa plants with the combined inoculation of Azospirillum lipoferum and Pantoea agglomerans had the maximum antioxidant potential, especially in petroleum ether extracts. In the methanolic extract, a higher amount of radical scavenging was observed as compare to positive and negative control. There was also increase in fat, moisture and carbohydrate contents of the combination inoculated plant.

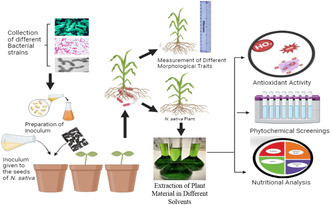

## 1 INTRODUCTION

Numerous cultures have used black cumin seeds (BCS), sometimes referred to as black seed or *N. sativa*, for both culinary and therapeutic purposes for a very long time. Their versatile applications in food‐ and nutrition‐related areas are supported by their rich nutritional composition and potential health benefits. Protein, fiber, vitamins, and minerals are all present in good amounts in black cumin seeds. They are particularly rich in calcium, magnesium, phosphorus, and iron. Additionally, they also contain small amounts of the vitamins A, C, and E (Afoakwah & Mahunu, 2023). Modern, intensive agriculture requires a high reliance on fertilizers and chemicals, which lead to pollution, environmental risks, and significant residual toxicity in products. In order to prevent the aforementioned issues, careful use of biofertilizers and plant growth regulators is now prioritized in order to boost growth, yield and nutrition, to obtain the highest profit per unit area, and to advance an environment‐friendly agricultural system (Pavankumar et al., 2018). The problem is absence of new agricultural techniques, lack of higher‐quality seeds, the failure to apply fertilizers at the recommended rates, and some issues with post‐harvest management are the main factors affecting the productivity and production of *N. sativa* (Haque et al., 2022). Numerous environmental, demographic, social, and institutional factors have affected the yield and production of black cumin in past (Teshome & Anshiso, 2019). Utilizing biostimulants is a significant approach to minimizing soil contamination, which is a crucial aspect of sustainable agriculture (Fawzy et al., 2012).

*Nigella sativa* is a crucial nutritional and pharmaceutical crop around the world; however, it is imported because it is a neglected crop in Pakistan (Aftab et al., 2022). Black cumin seeds are useful in traditional medicine for treating a variety of diseases including bronchitis, asthma, dizziness, nausea, chest congestion, dysmenorrhea, obesity, diabetes, paralysis, hemiplegia, back pain, infection, inflammation, rheumatism, hypertension, and gastrointestinal issues like dyspepsia, flatulence, dysentery, and diarrhea (Ara et al., 2020). *N. sativa* seeds provide many biological benefits, including anticancer, bacteriostasis, antihypertension, liver protection, anti‐inflammation, antipyretic, analgesia, and ROS removal. They are therefore frequently employed in food and medicine (Amin & Hosseinzadeh, 2015; Gholamnezhad et al., 2016). Herbs have been used by humans in all prehistoric cultures to heal illnesses and energize bodily systems (Ali et al., 2021). To lessen the risky effects of synthetic drugs, medicinal plants are now being considered valuable drug sources (Bahmani et al., 2014). Thymoquinone, a highly prevalent component of oil, possesses anticancer, immunomodulatory, antioxidant, anti‐inflammatory, and antihistaminic properties. Furthermore, numerous studies have demonstrated the diverse pharmacological properties of thymoquinone, which have been linked to its oil‐related properties. To assess the impact of *N. sativa* seeds on oxidative stress, inflammation, glycemic management, and other related topics, some clinical trials have been carried out on qualified patients (Hallajzadeh et al., 2020).

*Nigella sativa* L. is an annual plant, a member of the Ranunculaceae family, that reaches a height of 60–70 cm (Kıralan, 2014; Majeed et al., 2021). Farming season runs from November through April. Under optimum environmental conditions, seeds of this therapeutically significant herb require 25–30 days to germinate (Chaouche et al., 2014). *N. sativa* is also known as miracle plant and is considered “The herb from paradise” by early herbal experts (Ahmad et al., 2013). It is found mainly in Pakistan and India. This species is grown in Syria, Israel, Lebanon, Southern Europe, and Bangladesh (Naz, 2011; Paarakh, 2010). It has been reported that India produces 300–500 kg ha^−1^of crops (Giridhar et al., 2015). India, which produces about 1,939,000 tons, was the world's leading producer of spices, followed by Turkey (199,018 tons), China (113,359 tons), Pakistan (73,472 tons), Indonesia (110,387 tons), Bangladesh (180,993 tons), and Ethiopia, which produced around 36,754 tons (Dessie et al., 2020). It has been stated that Pakistan has a significant potential for the cultivation, propagation, and production of *N. sativa*, but very little for actual production (Rabbani et al., 2011).

Due to the issues brought about by the extensive use of chemical fertilizers in agricultural systems, the use of bio and organic inputs has recently increased. Among them, plant growth‐promoting rhizobacteria (PGPRs) are naturally occurring soil organisms that assist plants in obtaining sufficient nutrients and water by altering the physicochemical composition of the soil (Darakeh et al., 2021). The majority of the growth and yield metrics of numerous medicinal and aromatic plants increased to their maximum levels when phosphate‐solubilizing and nitrogen‐fixing bacteria were combined in the treatment (Hassan & Ali, 2013).

The biofertilizer *Azotobacter*, which contains N‐fixing bacteria from the genus *Azotobacte*r, is a molecularly selective N‐fixing substance that has the ability to synthesize active biological compounds in root zones, including auxins, gibberellins, biotin, pantothenic acid, nicotinic acid, etc. This aids in the formation of root systems (Ahmad et al., 2013). The free‐living microorganisms of the genus *Azospirillum* bacteria aid in the growth of *N. sativa* plants (PGPB) (Pii et al., 2015). *Azospirillum* produces siderophores, which control the crop's access to nutrients, and as a result, they play a significant role in yield‐related characteristics (Naderifar & Daneshian, 2012). One of the most extensively researched bacteria that promotes plant growth is *Azospirillum brasilense*. It is thought to be a free‐living soil bacterium that can influence the growth of many agricultural crops all over the world by excreting different hormones and having the capacity to fix nitrogen. Although the bacteria are thought to be free living, it seems to prefer being close to plant roots rather than open soil (Bashan et al., 2013). The current study was done in order to evaluate the bacteria‐base biofertilizer used to enhance the yield and nutritional potential of *N. sativa*.

Combining synthetic and biofertilizers to improve *N. sativa* plant growth is an innovative way to connect current technology with ancient farming practices. This integrated approach seeks to optimize crop yield and quality while minimizing environmental impact, providing a balanced and sustainable method for improving the growth and nutritional content of *N. sativa*. It is important because it represents a step forward in crop cultivation optimization for both environmental responsibility and productivity. Specifically, it improves agricultural practices toward resilience and eco‐friendliness.

## 2 MATERIALS AND METHODS

### 2.1 Study area and sample collection

The pot experiment was performed at the Botanical Garden of LCWU Lahore (longitude 31.545 °N and latitude 74.3272 °E). Sandy loamy soil was collected from the local nursery. The seeds of *N. sativa* were obtained from the Punjab Seed Corporation Center, and washed with distilled water before sowing seeds were soaked overnight for germination.

### 2.2 Presoil analysis

The soil sample was air dried and sieved to remove stones. Presoil analysis was performed to check soil texture, electrical conductivity (EC), potential of hydrogen (pH), organic matter (O.M.), CaCo_3_, phosphorus, and potassium (Abdel‐Aziez et al., 2014).

### 2.3 Collection and culturing of bacterial strains

Three different bacterial strains, that is, *Azospirillum brasilense* (FCBP‐SB‐0025), *Azospirillum lipoferum* (FCBP‐PB‐0434), and *Pantoea agglomerans* (FCBP‐PB‐0454), were collected from First Culture Bank of Pakistan (FCBP), Institute of Agricultural Sciences (IAGS), University of Punjab, Lahore, and pure cultures were prepared.

### 2.4 Media preparation

The bacteria were cultured through LBA (Luria–Bertani agar) media by following the methodology of Priyom et al. (2022).

### 2.5 Inoculum preparation

Broth media of different bacterial strains were prepared. Bacterial culture was employed for inoculum preparation. In conical flask, 200 mL of distilled water was added, and they were autoclaved for 1 h to disinfect them. With the aid of an inoculating needle, bacterial colonies were moved to conical flask and then put on the shaker for 3 days.

After shaking, spectrophotometer was used to determine the bacterial optical density at 600 nm. The optical density of *Azospirillum brasilense, Azospirillum lipoferum, and Pantoea agglomerans* was measured to be 1.217 × 10^7^ cells cm^−3^, 1.406 × 10^8^ cells cm^−3^, and 1.645 × 10^9^ cells cm^−3^, respectively. Seeds were soaked in inoculum for 15–20 min before sowing either individually or in combination form (Priyom et al., 2022).

### 2.6 Experimental design

Complete randomized experiment was designed. *N. sativa* L. seeds were inoculated using broth media. Each sample of pots was treated with different treatments along with positive and negative controls. T1: *Azospirillim brasilense* (A), T2: *Azospirillum lipoferum* (B), T3: *Pantoea agglomerans* (C), T4*: Azo. brasilense* + *Azo. lipoferum* (A + B), T5: *Azo. lipoferum* + P. *agglomerans* (B + C), T6: *P. agglomerans* + *Azo. brasilense* (C + A), T7: *Azo. brasilense* + *Azo. lipoferum* + *P. agglomerans* (A + B + C), T8: addition of nitrogen fertilizer (N), T 9: addition of phosphorus fertilizer (P), T10: nitrogen and phosphorus fertilizers in combination form (N + P), and T11: control.

### 2.7 Germplasm cultivation

Seeds (165) were sown (15 in each of 11 pots) from November 2022 to April 2023 at the Botanical Garden of Lahore College for Women University Lahore. Weeds are controlled manually. The temperature for seed germination was maintained at 20–25°C. Pot conditions were maintained accordingly (Ghafoor & Ahmad, 2003).

### 2.8 Morphological traits selection

The morphological traits that were studied included number of seedlings, plant height, the total number of branches, number of leaves, the total number of flowers, stamen numbers, number of follicles, width of follicles, length of follicles, and the total number of seeds produced per follicle (Aftab et al., 2020).

### 2.9 Extraction of plant material

The plant material (seeds) was macerated to obtain the plant extract. About 50 g seeds of *N. sativa* were soaked in 150 mL of petroleum ether for 4 days. After filtering the soaked seeds, the remaining plant material was soaked in chloroform for an additional 4 days. The seeds were filtered and then soaked in methanol for a further 4 days. In the end, filtered seeds were soaked in distilled water for next 4 days (Yessuf, 2015).

### 2.10 Qualitative phytochemical screening

The crude extract was examined for the presence of bioactive substances using the prescribed procedures (Kamal, 2014; Madhukar, 2013). Maceration of *N. sativa* seeds was dissolved in their respective solvents (petroleum ether, chloroform, methanol, and distilled water), and resulting solutions were used for in vitro analysis. Phytochemicals like alkaloids, tannins, flavonoids, steroids, and saponins were qualitatively analyzed.

### 2.11 Antioxidant assay

Analysis was performed under three different assays including total phenolic content, total antioxidant analysis, and DPPH free radical scavenging activity analysis following the methodology of Nadeem et al. (2022).

### 2.12 Total phenolic content

The total phenolic contents of plant extracts were calculated by following the methodology of Tian et al. (2021). The extract (0.1 mL) was added to 2.8 mL of 10% Na_2_CO_3_ and 0.1 mL of 2 N Folin–Ciocalteu (FC) reagent. The absorbance at 725 nm of the reaction mixture was determined by a UV–visible spectrophotometer. The total phenolic content was calculated from a gallic acid standard curve and represented as gallic acid equivalents (μg g^−1^ of dry weight) using the expression. Gallic acid equivalent standard calibration curve created using *y* = 0.005*x* + 0.047 and varying gallic acid concentrations (where *R* ^2^ = 0.998).

### 2.13 Total antioxidant analysis

Antioxidant capacity of plant extracts was determined by following the methodology of Aftab et al. (2020). The extracts (0.6 M sulfuric acid, 4 mM ammonium molybdate, and 28 mM sodium phosphate) were mixed with 1.9 mL of the reagent solution. After 60 min of incubation at 95°, the reaction mixture was allowed to cool to room temperature. The antioxidant activity of the material was expressed by measuring its absorption at 695 nm. Butyl hydroxytoluene (BHT) of 0.476 mg mL^−1^ was used as standard.

### 2.14 2, 2‐Diphenyl‐1‐picryl‐hydrazyl radical (DPPH) free radical scavenging

The reaction mixture was prepared by following the methodology of Pedan et al. (2016): DPPH solution was prepared in DMSO by taking 0.002 g of DPPH by dissolving in 120 mL DMSO. Both the plant extract and DPPH solution were added in each test tube in equal quantities. The mixture was mixed and then incubated for 30 min in the dark. The scavenging activity was measured as an absorption at 517 nm, and the entire process was carried out in triplicate. The scavenging activity of the DPPH radical (SC %) was used to measure the antioxidant activity.

$$\mathrm{SC}\%=\left[1-\left(\text{absorbance of sample}\right)/\left(\text{absorbance of control}\right)\right]\times 100$$

### 2.15 Nutritional analysis

The Association of Official Analytical Chemists (AOAC) methodologies were used to determine the proximate analysis of *Nigella sativa* (fats, carbohydrates, proteins, moisture, and ash). The Kjeldahl method was used to determine the protein content. The weight difference method was used to compute ash and moisture, and the soxhlet and solvent extraction methods were used to calculate fats (Javed, Shahid, et al., 2012; Javed, Shoaib, et al., 2012). The amount of protein, fat, moisture, and ash was subtracted from 100 to get the amount of carbohydrates in *Nigella sativa* seeds. To ensure the accuracy of the results, every analytical technique was carried out at least three times. The values were indicated as the mean ± standard deviation (Kaushik & Barmanray, 2022).

$$\text{Moisture}\;\left(\%\right)=\left(\text{initial weight}-\text{final weight}\right)/\text{weight of sample}\times 100$$

$$\begin{array}{c} \mathrm{Ash}\;\text{content}\;\left(g/100\;g\;\text{sample}\right)\\ \;=\text{weight of}\;\mathrm{ash}/\text{weight of sample taken}\times 100 \end{array}$$

$$\mathrm{Fat}\;\left(\%\right)=\text{weight of}\;\mathrm{dry}\;\text{flask}–\text{weight of empty flask}/10\times 100$$

$$\text{Carbohydrates}\;\left(\%\right)=100–\left(\text{Moisture}\%+\mathrm{Ash}\%+\mathrm{Fat}+\text{Protein}\%\right)$$

### 2.16 Statistical analysis

The mean ± standard deviation of the data was calculated. Two‐way ANOVA was used to analyze the effects of several bacterial strains in *N. sativa* L. using SPSS 2022 version 6.5. The IC_50_ values were calculated using Graph Pad 10.0.2.

### 2.17 Postsoil analysis

After harvest, the postsoil analysis was done by soil and water testing laboratory for research to check soil pH, OM (organic matter), N (nitrogen), K (potassium), and P (phosphorus) (Abdel‐Aziez et al., 2014).

## 3 RESULTS

### 3.1 Presoil analysis

The chemical parameters of soil included pH 8.01, EC 1.59 dS m^−1^, OM 0.48%, total CaCo_3_ 5.83%, and total P and K of 10.0 mg kg^−1^ and 121 mg kg^−1^, respectively, were analyzed. The physical properties of the soil were used, which included 50% sand, 45% silt, and 5% clay.

### 3.2 Morphological traits

**FIGURE 1:**
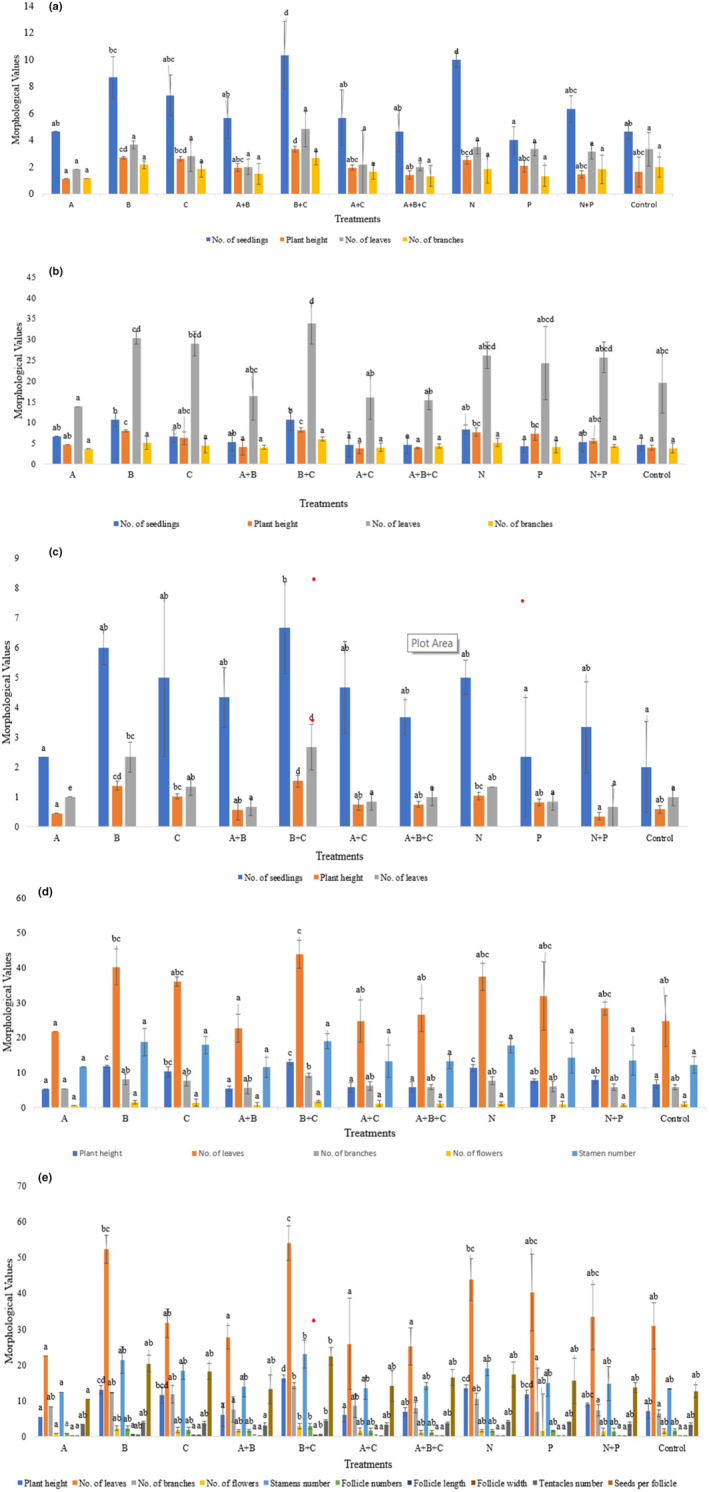
Morphological markers of *N. sativa* L. at (a) 15, (b) 3, (c) 60, (d) 90, and (e) 120 days interval.

*Nigella sativa* seeds were inoculated for 15–30 min before sowing. Days of germination were noted after sowing. After 2 weeks of sowing, complete germination of plantlets was observed. During the examination of 11 morphological traits, the number of germinated seedlings, plant height (PH), number of leaves (NL), total number of branches (NB), number of flowers (NF), stamen numbers (SN), follicle numbers (FN), follicle length (FL), follicle width (FW), tentacles numbers (TN), and seeds per follicle (S/F) were recorded. The seeds of *N. sativa* were treated with various bacterial inoculum and their morphological traits at different time intervals after 15 days, 30 days, 60 days, 90 days, and 120 days were recorded. As can be seen in Figure 1a, the maximum number of germinated seedlings was observed in treatment B + C (6.67 ± 1.53^b^), and a minimum number of seedlings was observed in the control plant. The highest plant height (cm) was measured in treatment number B + C (1.53 ± 0.19^d^) and the lowest plant height was measured in treatment N + P (0.35 ± 0.13^a^). A number of leaves were observed in treatment B + C (2.67 ± 0.29^d^) while a minimum number of leaves were observed in treatments A + B (0.67 ± 0.76^a^) and N + P (0.67 ± 0.29^a^).

The morphological traits at 30‐day intervals of *N. sativa* were calculated as shown in Figure 1b. A combination of B + C (*Azospirillum lipoferum + Pantoea agglomerans*) showed the maximum number of seedlings while treatment P showed the minimum number of seedlings. Maximum height (cm) among all these treatments was shown by B + C (3.33 ± 0.21^d^), whereas minimum was shown by treatment A (1.13 ± 0.08^a^). The maximum number of leaves was noted in treatment B + C (4.83 ± 2.57^a^) while minimum number of leaves was recorded in treatment A (1.83 ± 0.29^a^). The highest number of branches was recorded in B + C (2.67 ± 0.58^a^) in 11 treatments, whereas the lowest number of branches was noted in A (1.17 ± 0.29^a^).

After 60 days of germination, data were recorded as displayed in Figure 1c. Different morphological traits were calculated. Among all treatments, combination B + C showed maximum growth (10.67 ± 3.06^b^), highest plant height (8.2 ± 1.26^c^), largest number of leaves (33.83 ± 5.25^d^), and highest number of branches (6 ± 1^a^). The minimum number of plant growth in treatment P (4.33 ± 2.31^a^), the lowest plant height was show in treatment A + C (3.85 ± 0.15^a^), the smallest number of leaves were noted in treatment A (13.83 ± 1.53^a^), and the minimum number of branches in treatment P (3.67 ± 1.61^a^). Data were noted after 90 days of germination as displayed in Figure 1d. It was noted that B + C combination showed the maximum growth, plant height (13.01 ± 1.34^c^), number of leaves (43.83 ± 6.01^c^), number of branches (9.17 ± 1.26^b^), number of flowers (1.67 ± 0.76^a^), and stamen number (19 ± 4.58^a^). While the minimum values were noted, that is, 5.21 ± 0.38^a^ for plant height, 21.67 ± 5.13^a^ for number of leaves, and 5.33 ± 1.53^a^ for branches in treatment A while the minimum stamen number was noted in combination with A + B (11.5 ± 2.18^a^).

Data were analyzed after 120 days of germination as shown in Figure 1e. The morphological characters were calculated for maximum and minimum yield. Combination B + C was observed as the best treatment for plant growth and yield. It showed maximum height (16.32 ± 1.96^d^), the largest number of leaves (54 ± 12.77^c^), the highest number of branches (14.17 ± 3.21^b^), maximum flowers (2.83 ± 0.76^b^), largest stamen number (23 ± 3^b^), largest follicle number (2.83 ± 0.76^b^), follicle length and width (0.48 ± 0.16^a^ and 0.55 ± 0.05^b^), highest tentacles (4.33 ± 0.5^b^), and maximum yield as the maximum number of seeds per follicle (22.33 ± 4.37^b^). These results suggested that treatment combination B + C is the most effective for promoting plant yield. The reason for this is not entirely clear, but it is possible that the combination of treatments B and C provides the plants with the optimal balance of nutrients and environmental conditions. Treatment B + C (*Azospirillum lipoferum* + *Pantoea agglomerans*) and nitrogen both showed good results in morphological traits, which is why antioxidant analysis, phytochemical screenings, and nutritional analysis were done.

**FIGURE 2:**
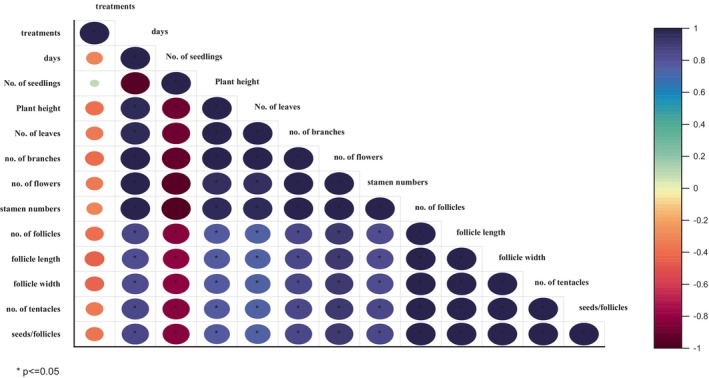
Correlation graph of different morphological traits of *N. sativa* L.

**FIGURE 3:**
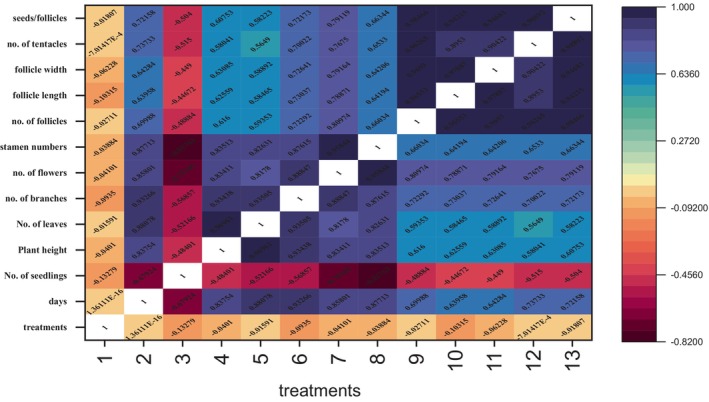
Heatmap of different morphological traits of *N. sativa* L.

On the basis of different morphological traits, the simple correlation coefficient was determined between 11 treatments and 11 morphological traits, and their average values by using Origin 2016 and Python. For this analysis, the following morphological traits were taken into account: number of seedlings, plant height, number of leaves, number of branches, number of flowers, stamen number, number of follicles, follicle length, follicle width, number of tentacles, and seed per follicle. The variable traits showed both positive and negative correlations. There was positive link between all of the variables, as shown by the positive correlation coefficients in Figure 2. However, the strength of the correlation varied among the different variables. All parameters showed nonsignificant relation to all treatments. The correlation coefficient between number of seedlings and all traits showed negative correlation. This indicated that there may be a slight tendency for plant height to increase when the number of seedlings declines. Numerous other factors in the study, such as the number of leaves, branches, flowers, and stamens, also have correlation coefficients indicating their links with other variables. Pearson correlation was done through Origin 2016 with Python as shown in Figure 2, and heatmap of different morphological traits was displayed in Figure 3.

### 3.3 Qualitative phytochemical screening

Based on morphological traits, treatment B + C (*Azospirillum lipoferum* + *Pantoea agglomerans*) and nitrogen produced the best growth rate and yield. Treatments B + C and the control were compared for further phytochemical screening and antioxidant and nutritional analyses.

#### 3.3.1 Alkaloids (Wagner's reagents)

An orange–yellow precipitate formed, which was an indication of alkaloids. Petroleum ether and chloroform extracts showed maximum concentration of alkaloids, whereas methanol showed less concentration of alkaloids. While in control, alkaloids were detected only in petroleum ether extract as can be seen in Table 1.

**TABLE 1:** Identification of phytochemical screenings in *N. sativa* L. through solvent extraction.

	Petroleum ether	Chloroform	Methanol	Distilled water
*Alkaloids*				
Treatment	+++	+++	++	−
Control	+++	−	−	−
*Tannins*				
Treatment	−	−	+++	++
Control	−	−	+	++
*Flavonoids*				
Treatment	+	++	+++	++
Control	−	++	+++	+
*Steroids*				
Treatment	+++	++	+	−
Control	−	+++	+++	−
*Saponins*				
Treatment	++	+++	−	−
Control	−	+++	−	−

*Note*: Key: +++ highly concentrated, ++ very concentrated, + concentrated, and − absent.

#### 3.3.2 Tannins

The formation of blue–green color indicated the presence of tannins. Methanol showed maximum concentration as compared to distilled water, whereas in case of control, tannins were identified in distilled water than methanol as displayed in Table 1.

#### 3.3.3 Flavonoids

A bright green color formed, which indicated the presence of flavonoids. The highest concentrations of flavonoids were examined in the order of methanol, petroleum ether, chloroform, and distilled water. In contrast with control, highest concentration of flavonoids was determined in following patterns: methanol > chloroform > distilled water > petroleum ether as shown in Table 1.

#### 3.3.4 Steroids

The interface turned reddish brown indicating the presence of steroids. The highest concentration of steroids was evaluated in following patterns, petroleum ether > chloroform > methanol > distilled water, as can be seen in Table 1, whereas in control, steroids were absent in distilled water and petroleum ether extracts. Chloroform and methanol showed best identification for steroid detection.

#### 3.3.5 Saponins

A foamy layer developed on the surface of reagent mixture, and saponins were detected. Chloroform extract exhibited more concentration of saponins than other extracts. The chloroform extract showed the higher amount of saponins as compared to control (Table 1).

### 3.4 Antioxidant assay

Antioxidant evaluation was done through DPPH assay, total antioxidant, and total phenolic content assays. Nitrogen was the most effective synthetic fertilizer which produced the highest yield. Therefore, treatment B + C and nitrogen were used for further analysis.

### 3.5 Total phenolic content

To determine the total phenolic content, absorbance was measured at the wavelength of 725 nm. Seven serial dilutions were prepared. The nitrogen treatment was used as the positive control. At the concentration of 200 mg mL^−1^ of dilutions, the maximum phenolic content value (275.11 ± 1.30^g^) was found in the petroleum ether extract of treatment B + C (Table 2), whereas at the concentration of 3.125 mg mL^−1^, the lowest value was observed as 50.56 ± 0.91^g^. Chloroform extract has its maximum absorbance value, 256.22 ± 1.07^f^ at 200 mg mL^−1^ concentration, while 46.28 ± 0.48^f^ was its minimum value at 3.125 mg mL^−1^ concentration. Methanol extract showed the same results, indicating that at the 200 mg mL^−1^ concentration, extracts indicated their maximum absorbance value, and at the 3.125 mg mL^−1^ concentration, extract value became minimal. At the 200 mg mL^−1^ concentration, the methanol extract showed its maximum absorbance value of 220.13 ± 0.83^d^, whereas the 3.125 mg mL^−1^ concentration yielded a minimum value of 47.45 ± 0.50^d^. Distilled water extract demonstrated 205.67 ± 0.79^b^ as maximum value at 200 mg mL^−1^ concentration while 44.22 ± 1.11^b^ at 3.125 mg mL^−1^ concentration (Table 2).

**TABLE 2:** Total phenolic content mg/mL estimation of different extracts at different concentrations of *N. sativa.*

Concentrations	Petroleum ether	Chloroform	Methanol	Distilled water
*Total phenolic content of N. sativa L*.				
200	275.11 ± 1.30^g^	256.22 ± 1.07^f^	220.13 ± 0.83^d^	205.67 ± 0.79^b^
100	213.67 ± 0.91^g^	223.1 ± 0.48^f^	198 ± 1^d^	177.07 ± 0.56^b^
50	185.33 ± 0.9^g^	179.21 ± 0.88^f^	175.24 ± 1.24^d^	148.71 ± 0.37^b^
25	146.23 ± 1.03^g^	145.87 ± 1.04^f^	139.69 ± 0.57^d^	115.17 ± 0.94^b^
12.5	93.11 ± 0.59^g^	88.11 ± 0.98^f^	85.33 ± 0.52^d^	77.81 ± 0.90^b^
6.26	69.24 ± 0.45^g^	59.48 ± 0.67^f^	65.44 ± 0.96^d^	63.06 ± 0.57^b^
3.125	50.56 ± 0.91^g^	46.28 ± 0.48^f^	47.45 ± 0.50^d^	44.22 ± 1.11^b^
Control	123.45 ± 1.39^c^	110.96 ± 1.34^a^	136.7 ± 1.87^e^	171 ± 1^h^

**FIGURE 4:**
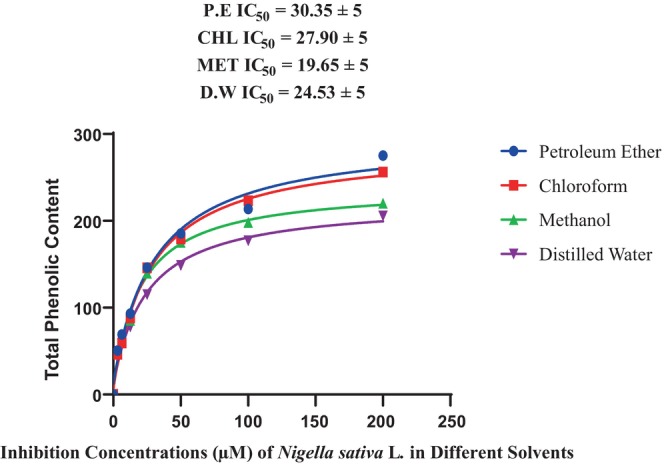
IC_50_ value of total phenolic content.

The IC_50_ value and degree of freedom of total phenolic content were highest in petroleum ether (30.35 ± 5 mg mL^−1^). The chloroform extract showed 27.90 ± 5 mg mL^−1^. IC_50_ value and degree of freedom. The IC_50_ value and degree of freedom in distilled water was 24.53 mg mL^−1^ while methanolic extract had lowest IC_50_ 19.65 ± 5 mg mL^−1^ (Figure 4).

### 3.6 Total antioxidant analysis

The standard value for all antioxidants was determined to be butyl hydroxytoluene (BHT), which is 0.476 mg mL^−1^. The plant extract's capacity to scavenge free radicals was compared to the control. The results of the study demonstrated that the antioxidant capacity of the extract varied depending on the solvent used. The maximum antioxidant capacity was found in petroleum ether extractions, followed by chloroform, methanol, and distilled water. Petroleum ether extractions at a 200 mg mL^−1^ concentration had the highest antioxidant capacity, ranging from 0.748 to 0.028^h^ in treatment B + C. In chloroform extractions, the highest antioxidant capacity was 0.643 ± 0.001^h^ at 200 mg mL^−1^ concentration, while minimum value was 0.031 ± 0.002^a^ at 3125 mg mL^−1^ concentration. However, the maximum antioxidant potential for methanol extraction was ranging from 0.517 ± 0.002^h^. The lowest antioxidant capacity was found in distilled water, which had a value of 0.431^h^ at a concentration of 200 mg mL^−1^. The least antioxidants were present in control (Table 3).

**TABLE 3:** Total Antioxidant estimation of different extracts at different concentrations of *N. sativa.*

Concentrations	Petroleum ether	Chloroform	Methanol	Distilled water
*Total antioxidant analysis*				
200	0.748 ± 0.002^h^	0.643 ± 0.001^h^	0.517 ± 0.002^h^	0.431 ± 0.002^h^
100	0.585 ± 0.001^f^	0.475 ± 0.001^f^	0.482 ± 0.003^f^	0.336 ± 0.001^f^
50	0.355 ± 0.004^e^	0.344 ± 0.01^e^	0.326 ± 0.002^e^	0.221 ± 0.002^e^
25	0.247 ± 0.002^d^	0.237 ± 0.002^d^	0.233 ± 0.002^d^	0.143 ± 0.002^d^
12.5	0.198 ± 0.002^c^	0.185 ± 0.001^c^	0.162 ± 0.001^c^	0.137 ± 0.002^c^
6.26	0.156 ± 0.002^b^	0.166 ± 0.001^b^	0.138 ± 0.001^b^	0.129 ± 0.002^b^
3.125	0.028 ± 0.001^a^	0.031 ± 0.002^a^	0.034 ± 0.001^a^	0.028 ± 0.001^a^
Control	0.702 ± 0.001^g^	0.632 ± 0.002^g^	0.465 ± 0.003^g^	0.251 ± 0.002^g^

**FIGURE 5:**
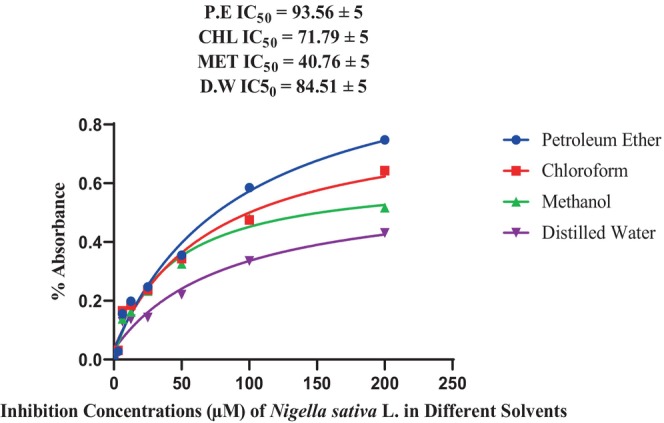
IC_50_ value of antioxidant assay.

The results of this study showed that the antioxidant levels of the solvents decreased along with the concentration. This indicates that when the solvents were more concentrated, they contained more antioxidants. The IC_50_ value and degree of freedom of total antioxidant analysis were highest in petroleum ether 93.56 ± 5 mg mL^−1^ and lowest in methanol 40.76 ± 5 mg mL^−1^. While chloroform has 71.79 ± 5 mg mL^−1^. IC_50_ value and distilled water displayed 84.51 ± 5 mg mL^−1^ (Figure 5).

### 3.7 2, 2‐Diphenyl‐1‐picryl‐hydrazyl radical (DPPH) free radical scavenging

The most popular method for determining a plant extract's ability to 2,2‐diphenyle‐1‐picryl‐hydrazyl (DPPH) free radical scavenging activity. The DPPH radical scavenging activity was measured by checking the absorbance at 517 nm using different concentrations. Each sample was tested three times to ensure accuracy. The ability of the plant extracts to neutralize free radicals was compared to control.

**FIGURE 6:**
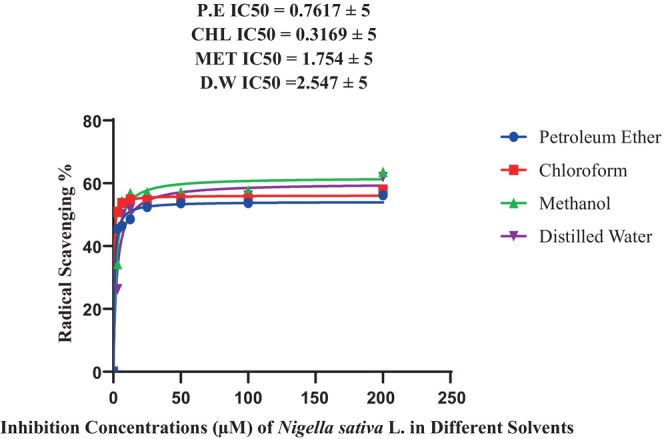
IC50 value of DPPH.

Treatment B + C (*Azo. lipoferum + P. agglomerans*) of methanol extraction generated the highest percentage (63.56 ± 1.99^de^) at a 200 mg mL^−1^ concentration as compared to earlier extractions. Distilled water indicated the maximum absorbance (61.99 ± 1.55^c^), while chloroform extraction showed the maximum value (57.96 ± 1.03^e^) at 200 concentrations. The petroleum ether extraction showed the lowest value, that is, 56.17 ± 1.89^cd^ in DPPH, and showed lowest value as compared to standard value as seen in Table 4. The IC_50_ value and degree of freedom of DPPH were 0.7617 ± 5 in petroleum ether, 0.3169 ± 5 in chloroform, 1.754 ± 5 in methanol, and 2.547 ± 5 in distilled water (Figure 6).

**TABLE 4:** 2, 2‐Diphenyl‐1‐Picryl‐Hydrazyl Radical (DPPH) free radical scavenging.

Concentrations	Petroleum ether	Chloroform	Methanol	Distilled water
*DPPH*				
200	56.17 ± 1.89^cd^	57.96 ± 1.03^e^	63.56 ± 1.99^de^	61.99 ± 1.55^c^
100	53.69 ± 1.92^cd^	55.10 ± 1.47^e^	57.61 ± 2.12^de^	56.11 ± 1.43^c^
50	53.63 ± 2.28^cd^	55.03 ± 0.31^e^	57.12 ± 0.97^de^	53.92 ± 1.20^c^
25	52.45 ± 12.15^cd^	54.65 ± 1.67^e^	57.07 ± 0.63^de^	53.48 ± 2.06^c^
12.5	48.57 ± 2.12^cd^	54.8 ± 1.27^e^	56.82 ± 1.85^de^	51.76 ± 1.85^c^
6.26	46.45 ± 1.49^cd^	53.67 ± 1.98^e^	54.35 ± 1.66^de^	50.12 ± 1.28^c^
3.125	45.43 ± 2.27^cd^	50.92 ± 1.64^e^	34.32 ± 1.60^de^	26.32 ± 2.32^c^
Control	51.63 ± 1.71^cde^	54.48 ± 1.26^e^	34.15 ± 2.56^b^	24.51 ± 3.90^a^

**FIGURE 7:**
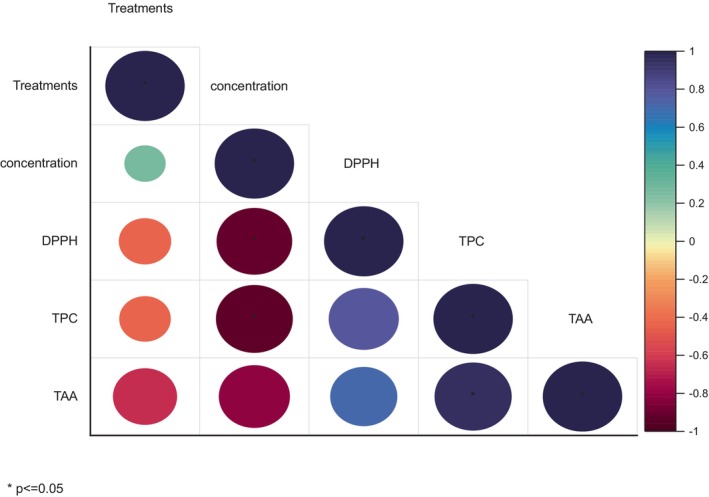
Correlation graph of antioxidant analysis.

**FIGURE 8:**
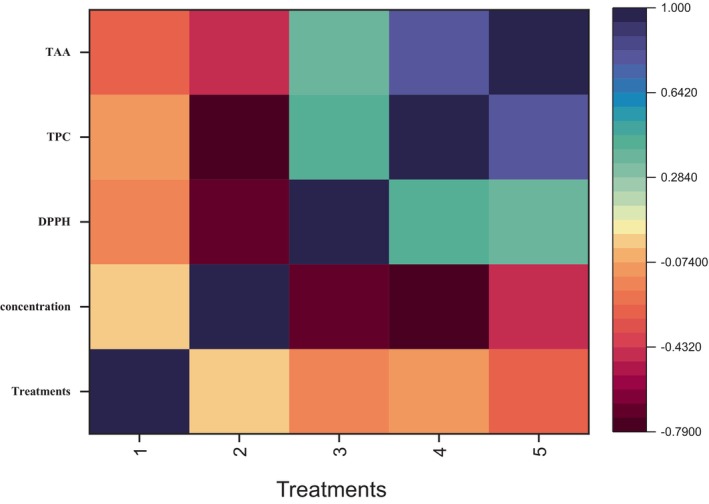
Heatmap of antioxidant activity.

Pearson correlation of all three antioxidant activities was performed through Origin 2016 and Python. Concentration and DPPH had a correlation value of −0.685. This indicated a weakly negative correlation between the antioxidant assay's concentration and DPPH findings. DPPH tends to decrease as the concentration increases. The correlation coefficient between TPC and TAA is 0.796, which shows a very strong positive correlation between the two variables. This showed that total antioxidant activity (TAA) tends to increase along with an increase in total phenolic content (TPC). Along with the significant level of the correlation coefficient, Figure 7 shows the correlation between the three antioxidant activities. The significant difference between the treatment B + C and control groups was examined using two‐way analysis of variance (ANOVA) on data from the TPC, DPPH, and TAC. *P* values <.05 showed statistically significant values. Heatmap of antioxidant activity is shown in Figure 8.

### 3.8 Nutritional analysis

Only the B + C treatment and control group were utilized for the proximal analysis.

### 3.9 Estimation of moisture

Petroleum ether, chloroform, methanol, and distilled water are the four distinct solvents used in the study to assess the quantities of various components. The percentage of moisture content contained in the sample is represented by the numbers shown for each solvent, along with their corresponding standard deviations.

The moisture content of petroleum ether was determined to be 95.19 ± 0.47^abc^ %. The moisture content of chloroform was slightly higher at 96.08 ± 0.53^bc^. The amount of moisture content in methanol was the greatest of the listed chemicals. The moisture content of methanol was 96.49^c^% with a standard variation of 0.74 and the moisture content of distilled water was 95.61 ± 0.62^abc^.

A control group is also included in the table for comparison. The reference values or baseline measurements are represented by the control group. Petroleum ether had a 94.44 ± 0.43^a^ moisture content in the control group. It was 95.45^abc^ ± 0.42 for the chloroform. Compared to distilled water, which had a moisture content of 95.14 + 0.28, methanol had a moisture level of 94.88^ab^, and a standard deviation of 0.8. The moisture contents of all the substances were somewhat higher than the control values, which ranged from 94.44 ± 0.43^a^ to 95.14 ± 0.28^abc^ (Table 5).

**TABLE 5:** Moisture content of different solvents.

	Petroleum ether	Chloroform	Methanol	Distilled water
*Nutritional analysis of moisture*				
Moisture	95.19 ± 0.47^abc^	96.08 ± 0.53^bc^	96.49 ± 0.74^c^	95.61 ± 0.62^abc^
Control	94.44 ± 0.43^a^	95.45 ± 0.42^abc^	94.88 ± 0.8^ab^	95.14 ± 0.28^abc^

### 3.10 Determination of Ash

The table displays the ash concentration of four distinct substances: petroleum ether, chloroform, methanol, and distilled water. The percentage of ash content was determined, and each value also includes the standard deviation (SD). The ash content of the other compounds was compared to the control group, which served as a reference group. The ash content of the control group was 0.15 ± 0.01^b^, which was somewhat less than the ash level of the other compounds. Petroleum ether was found to have an average ash concentration of 0.47^d^, with a standard deviation of 0.20. The results demonstrate that petroleum ether extract had higher amount of ash content as compared to other extracts. The least amount of ash was found in chloroform. This shows that the amount of ash in chloroform was very little or negligible. Methanol had a relatively lower ash content of 0.06^a^ with a standard deviation of 0.01. Ash level in distilled water was 0.20^c^, with a standard deviation of 0.01. In comparison to methanol and chloroform, this shows a larger ash level, but it is still lower than petroleum ether (Table 6).

**TABLE 6:** Ash content of different solvents.

	Petroleum ether	Chloroform	Methanol	Distilled water
*Nutritional analysis of ash*				
Ash	0.47 ± 0.20^d^	0.05 ± 0^a^	0.06 ± 0.01^a^	0.20 ± 0.01^c^
Control	0.15 ± 0.01^b^	0.04 ± 0.01^a^	0.05 ± 0.006^a^	0.05 ± 0.03^a^

### 3.11 Fat content

The fat content of several solvents, such as petroleum ether, chloroform, methanol, and distilled water, was investigated using a nutritional analysis. The proportion of fat content in each solvent is displayed in the following table, which summarizes the findings of this investigation.

It was found that petroleum ether contains 2.44^e^ fat. The lowest fat level among the solvents was found in methanol, which had a fat content of 1.02^ab^, while chloroform had a fat value of 1.49^abcd^. A 2^de^ fat content was present in distilled water.

A control group was analyzed for the purpose of comparison. The reference values for fat content were provided by the control measures. Petroleum ether had a fat level of 1.54^bcd^ in the control group, chloroform had a fat content of 1.35^abc^, and methanol from the control group had a 0.9^a^fat content. In the control group, distilled water had a fat level of 1.85^cde^ (Table 7).

**TABLE 7:** Fat content of different solvents.

	Petroleum ether	Chloroform	Methanol	Distilled water
*Nutritional analysis of fat content*				
Fat	2.44 ± 0.11^e^	1.49 ± 0.06^abcd^	1.02 ± 0.17^ab^	2 ± 0.55^de^
Control	1.54 ± 0.12^bcd^	1.35 ± 0.15^abc^	0.9 ± 0.07^a^	1.85 ± 0.17^cde^

### 3.12 Estimation of protein

Petroleum ether was found to contain 1.34 ± 0.06^ab^ protein. Methanol had a greater protein content than chloroform (1.95 ± 0.60^b^), which had a slightly lower protein level of 1.87 ± 0.07^b^. The protein level of distilled water was 1.66 ± 0.02^b^.

An analytical control group was used to create a baseline for comparison. The reference values for protein content were provided by the control measurements. Petroleum ether had 1.33 ± 0.01^ab^ protein content in the control group. A bit higher protein concentration of 1.40 ± 0.07^ab^ was seen in chloroform. Methanol from the control group had 0.85 ± 0.12^a^ protein. In the control group, distilled water had a protein level of 1.59 ± 0.1^b^ (Table 8).

**TABLE 8:** Protein content of different solvents.

	Petroleum ether	Chloroform	Methanol	Distilled water
*Nutritional analysis of protein content*				
Protein	1.34 ± 0.06^ab^	1.87 ± 0.07^b^	1.95 ± 0.60^b^	1.66 ± 0.02^b^
Control	1.33 ± 0.01^ab^	1.40 ± 0.07^ab^	0.85 ± 0.12^a^	1.59 ± 0.1^b^

### 3.13 Carbohydrate content

Petroleum ether was found to contain 0.57 ± 0.34^a^ carbohydrates. Methanol has a somewhat lower carbohydrate content than chloroform (0.48 ± 0.08^a^ vs. 0.51 ± 0.41^a^). Carbohydrate content in distilled water was 0.52 ± 0.14^a^. The baseline values for the carbohydrate content were represented by the control measures. Petroleum ether had 2.52 ± 0.42^bc^ carbohydrate content in the control group. A somewhat lower carbohydrate percentage of 1.76 ± 0.22^b^ was seen in chloroform. The control group's methanol had a higher carbohydrate content of 3.33 ± 0.65^c^. The control group's distilled water contained 1.38 ± 0.02^ab^ carbohydrate content (see Table 9).

**TABLE 9:** Carbohydrate content in different solvents.

	Petroleum ether	Chloroform	Methanol	Distilled water
*Nutritional analysis*				
Carbohydrates	0.57 ± 0.34^a^	0.51 ± 0.41^a^	0.48 ± 0.08^a^	0.52 ± 0.14^a^
Control	2.52 ± 0.42^bc^	1.76 ± 0.22^b^	3.33 ± 0.65^c^	1.38 ± 0.02^ab^

### 3.14 Postsoil analysis

Postsoil analysis was done by soil and water testing laboratory for research. The results demonstrated that sandy loamy soil was used, EC (1.27 mS cm^−1^), pH (7.78), O.M (0.56%), available phosphorus (8.7 mg kg^−1^), available potassium (105 mg kg^−1^), and saturation was 38%.

## 4 DISCUSSION

These biofertilizers are capable of providing the plant with vital nutrients like nitrogen, phosphorus, and potassium, which can aid in enhancing plant growth and development. Additionally, they may support the suppression of pests and plant diseases, which could increase crop productivity. The agriculture research sector is focused on enhancing the production of crops through the application of biofertilizers and synthetic fertilizers.

*Nigella sativa* L. is a valuable plant in traditional medicine and is well known for its culinary applications. Black cumin is under cultivation in various parts of the world including Egypt, Iran, Greece, Syria, Albania, Turkey, Saudi Arabia, and India, but negligible in Pakistan, being climate sensitive. It is native to a large area of the eastern Mediterranean, northern Africa, the Indian subcontinent, and Southwest Asia. Black cumin, considered a panacea, has been recommended in traditional medicine in the forms of essential oil, paste, powder, and extract for a wide range of illnesses and conditions, including eczema, anorexia, amenorrhea, hypertension, rheumatism, headache, and back pain (Chaudhry et al., 2020). United Nation Sustainable Goal 15 (life on land) and UN Sustainable Goal 3 (excellent health and well‐being) both were accomplished through the production of *N. sativa* in Pakistan. There is a lot of study on *N. sativa* cultivation in Pakistan, however, it has not been conducted on a large scale. Most of the kalonji that is consumed in Pakistan is imported from other countries (Rabbani et al., 2011).

Due to the problem induced by the heavy use of chemical fertilizers in agricultural systems, the use of bio and organic inputs has been increasing recently. Among them, plant growth‐promoting rhizobacteria (PGPRs) are naturally occurring soil bacteria that promote plants to obtain sufficient nutrients and water by altering the physicochemical characteristics of the soil. Rhizobacteria that encourage plant growth enhance the physiological and biochemical processes of plants (Kumar et al., 2022). According to reports from *Mentha piperita* (del Rosario Cappellari et al., 2019), *Cannabis sativa* (Pagnani et al., 2018), and *Solanum lycopersicum* (Calvo‐Polanco et al., 2016), PGPRs have improved plant growth, secondary metabolism, and nutritional value. The mechanisms of action achieved by PGPR include biofilm formation for better soil binding to roots and maintaining relative water content, enhancement in nutrient uptake, triggering of osmotic response via production and accumulation of osmolytes, maintenance of ion homeostasis, enhancement in the production of plant growth regulators, improved antioxidant defense, chlorophyll, carbon, and nitrogen levels, and HCN production, in addition controlling the expression of proteins and genes that reduce stress (Bhat et al., 2023).

The majority of PGPRs can solubilize phosphate, increasing the quantity of phosphate ions that are accessible in the soil for plant absorption (Herrera Paredes & Lebeis, 2016). The enhancement of mineral nutrients by PGPRs in several plant species has been reported, which is consistent with our findings (Jang et al., 2017; Tinna et al., 2020). This enhancement could also be attributed to the rhizosphere's organic acid synthesis by bacteria and plants, which lowers soil pH and increases P, Ca, Fe, and Mn availability (Orhan et al., 2006).

According to the analysis, the increase in yield‐related parameters may be attributable to the combined effects of PGPR, *Azospirillum*, a nitrogen‐fixing organism that fixes free nitrogen and increases nitrogen uptake in plants, *Bacillus megaterium*, a phosphate‐solubilizing bacterium that increases phosphorous availability, and *Pseudomonas fluorescence*, a biocontrol agent used against pathogens that lessens the severity of biological stress. These results in black cumin are also supported by Ali and Hassan (2014) as well as Valadabadi and Farahani (2011).

*Azospirillum lipoferum* and *Pantoea agglomerans* can help to improve crop yields by improving the nutrient uptake and water use efficiency of plants. They can also help plants to resist a variety of stresses, such as drought, salinity, and heat. Additionally, they can help to reduce the need for chemical fertilizers and pesticides, which can be harmful to the environment. The reason behind this success could include factors like nutrient availability, enhanced root development, and potentially other mechanisms that support plant growth (Bhat et al., 2023). *Azospirillum lipoferum* and *Pantoea agglomerans* used in combination may aid in reducing the development of *Fusarium oxysporum*, a fungus that can cause wilt disease in *N. sativa* plants.

The secretion of phytohormones (such as auxins, gibberellins, cytokinin, and nitric oxide) emitted by *Azospirillum* spp. could be a possible reason for increases in the growth and development of *N. sativa* L. (Cecagno et al., 2015).

The application of chemical fertilizer improved the N, K, and P concentrations in plants. NPK fertilizer increases rhizosphere availability so that plants can adequately absorb these vital components. Soils frequently suffer from a lack of essential nutrients in the majority of Iran. The application of soil amendments, which alter the soil's physical qualities and the availability of mineral nutrients, is the approach used to establish a suitable habitat for associated plants in situations where soils are insufficient (Olad et al., 2018).

The conventional extraction technique for obtaining phenolic chemicals from plant sources was maceration. Total antioxidant capacity (TAC), which includes TPC as a crucial component, is widely employed to evaluate antioxidant extracts. One can estimate how many phenolic compounds are present in the extract using the Folin‐Ciocalteu (FC) phenol reagent. It is abundantly obvious that different solvents separate and extract the components of black cumin with varying degrees of effectiveness (Mariod et al., 2009).

The phytochemical composition, antioxidant assay, and proximate analysis of seeds from *N. sativa* L. were all determined using different extracts. Using the best approaches or techniques, medicinally active components of interest from *N. sativa* L. seeds were successfully extracted, recognized, and qualified using a variety of solvents (petroleum ether, chloroform, methanol, and distilled water). The phytochemical characterization and antibacterial activity of *N. sativa* seeds were reported by Shafodino et al. (2022) utilizing several plant extracts, including petroleum spirit, ethyl acetate, methanol, and aqueous extract.

The presence of physiologically active chemicals in plants is mostly discovered by phytochemical screening of medicinal plants. Phytochemicals, which are secondary components, give plants their color, flavor, and natural defense against pests. Alkaloids, carboxylic acid, coumarins, phenol, resin, saponins, and steroid belong to the phytochemicals found in both *N. sativa* seeds and *Cassia angustifolia* leaves when they are extracted in various solvents such as petroleum ether, methanol, and distilled water. *N. sativa* and *Cassia angustifolia* solvent extracts contain high concentrations (+++) of alkaloids that are extracted in petroleum ether, methanol, and distilled water (Reddy et al., 2018). Alkaloids are a group of nitrogen‐containing substances with strong odors (Dey et al., 2020). Alkaloids are nonpolar compounds, so they are more soluble in petroleum ether and chloroform than in methanol, which is a polar solvent. Alkaloids are nonpolar compounds because they do not have any polar functional groups. Methanol is a polar solvent because it has an ‐OH group, which can form hydrogen bonds. Therefore, alkaloids are more soluble in petroleum ether and chloroform than in methanol.

Flavonoids were identified in significant concentrations in the *N. sativa* seeds extract with petroleum ether and distilled water (+++), while they were not present at all in the methanolic extract (−). The flavonoids in a petroleum ether extract of *Cassia angustifolia* leaf were not negative (−), but they were highly concentrated (+++) in the methanol and distilled water extracts of the same plant (Reddy et al., 2018). Other phytochemicals like flavonoids, phenols, and tannins were only found in methanol and aqueous extracts (Shafodino et al., 2022). Flavonoids are typically soluble in polar solvents, such as methanol. Petroleum ether is a nonpolar solvent, so it does not dissolve flavonoids. The study also found that the concentration of flavonoids in plant extracts can vary depending on the plant species, the part of the plant used, and the extraction method used. The study concluded that methanol is the best solvent for extracting flavonoids from plant materials. Flavonoids are more soluble in acidic solutions than in alkaline solutions.

Antioxidant activities were associated with total phenolic and flavonoid content, which were measured as rutin equivalents and pyrocatechol equivalents, respectively. The fraction with 40% ethanol had the highest total phenolic content (138.85 ± 0.53 mg PE/g extract), according to the results. Additionally, the fraction containing 20% ethanol showed the best antioxidant activity (IC50 = 1.26 ± 0.21 μg/mL) and the highest content of flavonoids (140.11 ± 5.47 mg RU/g extract) in the DPPH experiment. Antioxidant activity showed a strong correlation with both total flavonoid and total phenolic levels (Ahmed et al., 2014).

Many plants include a type of polyphenolic compound known as tannins (Tong et al., 2022). Polyphenolic substances include flavonoids, phenols, and tannins. Polar solvents, including methanol and water, are often able to dissolve polyphenolic compounds. In nonpolar solvents like petroleum ether and chloroform, they are less soluble (Shafodino et al., 2022). In current study, flavonoids are soluble in polar solvents, such as methanol. Water is also a polar solvent, but it is not as effective at extracting tannins as methanol.

*Nigella sativa* was shown to possess phenols, saponins, alkaloids, steroids, and terpenoids in the phytochemical investigation conducted by Desai et al. (2015). Several researchers (Ishtiaq et al., 2013; Kazemi, 2014; Saleh et al., 2018; Yessuf, 2015) reported similar findings. Many plants contain a class of glycosides known as saponins (Singh & Kaur, 2018). They have a long, hydrophobic tail that is soluble in oil and a short, hydrophilic head that is soluble in water (Wang et al., 2014). The chloroform extract showed more concentration of saponins as compared to other extracts. In petroleum ether and chloroform, steroids were mostly found soluble. In contrast to pure water, they were soluble in methanol as well. This may be due to the fact that steroids are nonpolar substances, and nonpolar solvents work better to dissolve nonpolar substances.

Phenolic acids are an important group of secondary metabolites that are present in medicinal plants and have potent antioxidant properties because of their carboxyl and hydroxyl groups (Cai et al., 2004). The antioxidant potential of plants is positively associated with their phenolic content (Kheiry et al., 2016). Antioxidants from plants can prevent numerous illnesses, including most malignancies and cardiovascular problems. Therefore, increasing the antioxidant activity of plants may play a key role in avoiding diseases that affect humans (Uttara et al., 2009).

The overall phenolic content decreased with increasing solvent polarity, with the solvents including petroleum ether, chloroform, methanol, and distilled water in that sequence. Since flavonoids are typically soluble in polar solvents and the seeds' bioactive components are attracted to the polar solvents, it was generally assumed that the TPC would increase with solvent polarity (Amina, 2016; Attree et al., 2015). However, it might not appear to be exactly the same as in this case, which is most likely due to the polyphenol profile and behavioral traits of each component. Fortunately, it is crucial to emphasize that solvents can be used to successfully extract *N. sativa* L. seeds' abundant phenolic components.

The variation in the percentage of phytoconstituents extracted in different solvents may be the cause of the extracts' differing scavenging capacities (Pavithra & Vadivukkarasi, 2015). Each extract's pattern closely resembled that of traditional BHT. Some of the extracts were significantly more potent scavengers than the standard BHT. Due to their ability to combat free radicals, antioxidants are crucial for the treatment of disease (Sylvie et al., 2014). Methanol is a polar solvent, which is why it had the highest scavenging percentage at the highest concentration in the extract. Antioxidants are examples of polar chemicals that are better dissolved by polar solvents. With decreasing concentrations of dilutions, the extracts' scavenging percentage was also reduced. This could be due to the fact that, according to Alrashidi et al. (2022), the concentration of dilutions reduces as the level of antioxidants in the extract decreases.

The total quantity of phenol in extracts and the total amount of antioxidants are significantly correlated (Chaouche et al., 2014). Extracts from petroleum ether are rich in antioxidants. Petroleum ether is frequently used to extract nonpolar chemicals from plant material, such as terpenes and steroids. These substances have antioxidant qualities, which means they can aid in preventing cell deterioration brought on by free radicals.

Carbohydrates are listed first, followed by protein, and then lipids, in decreasing order of dietary content which reported 5.4% moisture, ash 4.34%, carbohydrates 39% protein 24.05%, and fat 21.67% (Javed, Shahid, et al., 2012; Javed, Shoaib, et al., 2012). The nutritional analysis of *N. sativa* L. (carbohydrate, lipids, protein, moisture, and ash) was conducted using (Aoac, 2005) methodology. In this study, the average moisture content in back cumin (95.84 ± 0.12) was higher than the literature's mean value (7.12), which was based on research done in Bangladesh (Kabir et al., 2019), and the (4.2 ± 0.3) value published by Mamun and Absar (2018). According to Tura et al. (2023), the ash content is a measure or reflection of the amount of minerals in the food item. In Pakistan, a proximate examination of three distinct black cumin seed varieties (BS01, BS02, and BS03) revealed that their nutritional composition ranged from 4.28 to 4.72% for crude ash (Ahood et al., 2019). This study's average ash concentration in black cumin (0.20%) was lower than the (7.39) as reported by Kabir et al. (2019), and the study's (40.3) report from Ethiopia in Mamun and Absar (2018). As a result, there was a significant difference between the ash content in this investigation and the literature mean value. When the crude fat content of this study was compared to various values reported in the literature, the average mean values of the four different solvent extracts of black cumin (1.74%) were lower than the value of the mean fat content in black cumin (32.12%) reported by Tura et al. (2023) studied in Ethiopia.

Protein is crucial for healthy development, general body repair, and the production of hormones, enzymes, and other elements needed for optimal bodily function (Tura et al., 2023). The average black cumin crude protein concentration across all extracts is 1.71 ± 0.28, which is lower than the previously reported values of 18.9 ± 0.82 (Ali et al., 2015) and 28.0 ± 0.36 (Mamun & Absar, 2018). In black cumin, the overall mean value for carbohydrates in this study was 0.52 ± 0.16%, which was lower than the mean value reported in the literature (19.7% and 30%) (Kabir et al., 2019; Mamun & Absar, 2018). Subsequently, there was an immense variation between the mean value in the literature and the proximal contents in this experiment. According to Tura et al. (2023), this could be caused by changes in the meteorological, geographic, topographical, and mineral content of the crop region.

The two‐way ANOVA statistical analysis performed on mean values of replication of the zones of inhibition of various extracts and/or oils of *N. sativa* seeds revealed that the majority of these sources of variation (i.e., the type of extractant used, extraction technique employed, the type of pathogenic bacteria strains used, and variation due to error) were confirmed, and the results showed that they were highly significant at *p* < .05 (Shafodino et al., 2022). For statistical analysis, two‐way ANOVA was performed on the replicate's values of morphological traits, antioxidant assay, and nutritional analysis. The results revealed that they were highly significant at *p* < .05 and the values greater than *p* > .05 mean they were nonsignificant.

It would be important to deal with these difficulties in order to encourage the cultivation of kalonji in Pakistan. This includes starting projects of research to investigate its farming methods, performing tests to confirm its potential health advantages, providing farmers with training and support, developing awareness campaigns, and building a market for the crop's products.

## 5 CONCLUSION

In a recent study, the application of the combination of *Azospirillum lipoferum* and *Pantoea agglommerans* greatly improved numerous morphophysiological properties of kalonji plants. Notably, total phenolic content, TAA, and DPPH assays revealed that *Azospirillum lipoferum* and *Pantoea agglommerans* demonstrated excellent antioxidant capacity. Results revealed that the combination of *A. lipoferum* and *P. agglommerans* had a substantial impact on the nutritional analysis and phytochemicals in different solvent extracts. These findings emphasize that in order to increase kalonji production in Pakistan, several challenges must be addressed, including research into cultivating methods, validation of health benefits, farmer training, awareness campaigns, and creating a market for kalonji byproducts. For Kalonji production, effective collaboration among agricultural researchers, governmental organizations, NGOs, and farmers is essential.

## AUTHOR CONTRIBUTIONS

**Nayyab Naeem:** Investigation (equal). **Arusa Aftab:** Supervision (equal). **Humaira Rizwana:** Funding acquisition (equal). **Zill‐e‐Huma Aftab:** Methodology (equal). **Zubaida Yousaf:** Methodology (equal); resources (equal). **Zainab Maqbool:** Data curation (equal). **Zainab Shahzadi:** Writing‐review and editing (equal). [Corrections added 29 August 2024, after online publication: The author contributions of Zainab Shahzadi were added and Najat A. Bokhari were removed.]

## ACKNOWLEDGMENTS

In this research work special acknowledgement goes towards Research Supporting Project (number RSPD2024R1048), King Saud University, Riyadh, Saudi Arabia.

## FUNDING INFORMATION

To compile this project, funding was given by King Saud University, Saudi Arabia Researchers Supporting Project number (RSPD2024R1048).

## CONFLICT OF INTEREST STATEMENT

The authors confirm that they have no conflicts of interest.

## ETHICAL APPROVAL

This study does not involve any human or animal testing.

## Data Availability

Data will be provided upon reasonable request to corresponding author.

## References

[fsn33982-bib-0001] Abdel‐Aziez, S. M. , Eweda, W. E. , Girgis, M. , & Ghany, B. F. A. (2014). Improving the productivity and quality of black cumin (*Nigella sativa*) by using Azotobacter as N_2_ biofertilizer. Annals of Agricultural Sciences, 59(1), 95–108.

[fsn33982-bib-0002] Afoakwah, N. A. , & Mahunu, G. K. (2023). Nutritional, biochemical, and functional characteristics of black cumin seeds. In Biochemistry, nutrition, and therapeutics of black cumin seed (pp. 27–41). Academic Press.

[fsn33982-bib-0003] Aftab, A. , Yousaf, Z. , Aftab, Z.‐e.‐H. , Younas, A. , Riaz, N. , Rashid, M. , Shamsheer, H. B. , Razzaq, Z. , & Javaid, A. (2020). Pharmacological screening and GC‐MS analysis of vegetative/reproductive parts of *Nigella sativa* L. Pakistan Journal of Pharmaceutical Sciences, 33(5), 2103–2111. 33824119

[fsn33982-bib-0004] Aftab, A. , Yousaf, Z. , Rasheed, M. , Younas, A. , Qamar, N. R. , Yasin, H. , & Shamsher, H. B. (2022). Morphological variability assessment of worldwide germplasm of pharmaceutically important plant *Nigella Sativa* L. Jordan Journal of Pharmaceutical Sciences, 15(1), 82–106.

[fsn33982-bib-0005] Ahmad, A. , Husain, A. , Mujeeb, M. , Khan, S. A. , Najmi, A. K. , Siddique, N. A. , Damanhouri, Z. A. , & Anwar, F. (2013). A review on therapeutic potential of *Nigella sativa*: A miracle herb. Asian Pacific Journal of Tropical Biomedicine, 3(5), 337–352. 23646296 10.1016/S2221-1691(13)60075-1 PMC3642442

[fsn33982-bib-0006] Ahmed, A. , Khalid, N. , Ahmad, A. , Abbasi, N. A. , Latif, M. S. Z. , & Randhawa, M. A. (2014). Phytochemicals and biofunctional properties of buckwheat: A review. The Journal of Agricultural Science, 152(3), 349–369.

[fsn33982-bib-0008] Ahood, K. , Shahid, B. , Khalil, A. A. , Faiz‐ul‐Hassan, S. , Khan, A. A. , Khan, M. A. , Gull, H. , Aslam, A. , Shahid, Q. , Riaz, A. , & Amna, B. (2019). Varietal comparison of proximate analysis and mineral composition of Black cumin seed powder. Pakistan Journal of Food Sciences, 29(2), 5–9.

[fsn33982-bib-0009] Ali, A. , Waly, M. I. , Bhatt, N. , & Al‐Saady, N. A. (2015). Proximate and phytochemical composition and antioxidant properties of indigenous landraces of Omani fenugreek seeds. African Journal of Traditional, Complementary, and Alternative Medicines, 12(2), 149–154.

[fsn33982-bib-0010] Ali, E. , & Hassan, F. (2014). Bio‐production of *Nigella sativa* L. seeds and oil in Taif area. International Journal of Current Microbiology and Applied Sciences, 3(1), 315–328.

[fsn33982-bib-0011] Ali, H. , Alkowni, R. , Jaradat, N. , & Masri, M. (2021). Evaluation of phytochemical and pharmacological activities of *Taraxacum syriacum* and *Alchemilla arvensis* . Jordan Journal of Pharmaceutical Sciences, 14(4), 457–472.

[fsn33982-bib-0555] Alrashidi, M. , Derawi, D. , Salimon, J. , & Yusoff, M. (2022). The effects of different extraction solvents on the yield and antioxidant properties of Nigella sativa oil from Saudi Arabia. Journal of Taibah University for Science, 16(2), 330–336.

[fsn33982-bib-0012] Amin, B. , & Hosseinzadeh, H. (2015). Black cumin (*Nigella sativa*) and its active constituent, thymoquinone: An overview on the analgesic and anti‐inflammatory effects. Planta Medica, 82, 8–16. 26366755 10.1055/s-0035-1557838

[fsn33982-bib-0013] Amina, B. (2016). Toxicity and anti‐oxidant activity of the essential oil of *Nigella sativa* . Der Pharmacia Lettre, 8, 245–249.

[fsn33982-bib-0014] Aoac, C. A. (2005). Official methods of analysis of the Association of Analytical Chemists International. Official Methods.

[fsn33982-bib-0015] Ara, I. , Maqbool, M. , Fekadu, G. , Hajam, T. A. , & Dar, M. A. (2020). Pharmaceutical significance of *Nigella sativa* L., a wonder herb. Journal of Applied Pharmaceutical Sciences and Research, 3(4), 4–13.

[fsn33982-bib-0016] Attree, R. , Du, B. , & Xu, B. (2015). Distribution of phenolic compounds in seed coat and cotyledon, and their contribution to antioxidant capacities of red and black seed coat peanuts (*Arachis hypogaea* L.). Industrial Crops and Products, 67, 448–456.

[fsn33982-bib-0017] Bahmani, M. , Shirzad, H. , Majlesi, M. , Shahinfard, N. , & Rafieian‐Kopaei, M. (2014). A review study on analgesic applications of Iranian medicinal plants. Asian Pacific Journal of Tropical Medicine, 7, S43–S53. 10.1016/S1995-7645(14)60202-9 25312163

[fsn33982-bib-0018] Bashan, Y. , Kamnev, A. A. , & de Bashan, L. E. (2013). Tricalcium phosphate is inappropriate as a universal selection factor for isolating and testing phosphate‐solubilizing bacteria that enhance plant growth: A proposal for an alternative procedure. Biology and Fertility of Soils, 49(4), 465–479.

[fsn33982-bib-0019] Bhat, M. A. , Mishra, A. K. , Jan, S. , Bhat, M. A. , Kamal, M. A. , Rahman, S. , & Jan, A. T. (2023). Plant growth promoting rhizobacteria in plant health: A perspective study of the underground interaction. Plants, 12(3), 629. 36771713 10.3390/plants12030629 PMC9919780

[fsn33982-bib-0020] Cai, Y. , Luo, Q. , Sun, M. , & Corke, H. (2004). Antioxidant activity and phenolic compounds of 112 traditional Chinese medicinal plants associated with anticancer. Life Sciences, 74(17), 2157–2184. 14969719 10.1016/j.lfs.2003.09.047 PMC7126989

[fsn33982-bib-0021] Calvo‐Polanco, M. , Sánchez‐Romera, B. , Aroca, R. , Asins, M. J. , Declerck, S. , Dodd, I. C. , Martínez‐Andújar, C. , Albacete, A. , & Ruiz‐Lozano, J. M. (2016). Exploring the use of recombinant inbred lines in combination with beneficial microbial inoculants (AM fungus and PGPR) to improve drought stress tolerance in tomato. Environmental and Experimental Botany, 131, 47–57.

[fsn33982-bib-0022] Cecagno, R. , Fritsch, T. E. , & Schrank, I. S. (2015). The plant growth‐promoting bacteria *Azospirillum* amazonense: Genomic versatility and phytohormone pathway. BioMed Research International, 2015, 898592. 25866821 10.1155/2015/898592 PMC4383252

[fsn33982-bib-0023] Chaouche, T. M. , Haddouchi, F. , Ksouri, R. , & Atik‐Bekkara, F. (2014). Evaluation of antioxidant activity of hydromethanolic extracts of some medicinal species from South Algeria. Journal of the Chinese Medical Association, 77(6), 302–307. 24613372 10.1016/j.jcma.2014.01.009

[fsn33982-bib-0024] Chaudhry, Z. , Khera, R. A. , Hanif, M. A. , Ayub, M. A. , & Sumrra, S. H. (2020). Cumin. In Medicinal plants of South Asia (pp. 165–178). Elsevier.

[fsn33982-bib-0025] Darakeh, S. A. S. S. , Weisany, W. , Diyanat, M. , & Ebrahimi, R. (2021). Bio‐organic fertilizers induce biochemical changes and affect seed oil fatty acids composition in black cumin (*Nigella sativa* Linn). Industrial Crops and Products, 164, 113383.

[fsn33982-bib-0026] del Rosario Cappellari, L. , Santoro, M. V. , Schmidt, A. , Gershenzon, J. , & Banchio, E. (2019). Induction of essential oil production in Mentha × piperita by plant growth promoting bacteria was correlated with an increase in jasmonate and salicylate levels and a higher density of glandular trichomes. Plant Physiology and Biochemistry, 141, 142–153. 31163341 10.1016/j.plaphy.2019.05.030

[fsn33982-bib-0027] Desai, S. D. , Saheb, S. H. , Das, K. K. , & Haseena, S. (2015). Phytochemical analysis of *Nigella sativa* and it's antidiabetic effect. Journal of Pharmaceutical Sciences and Research, 7(8), 527.

[fsn33982-bib-0028] Dessie, A. B. , Abate, T. M. , Adane, B. T. , Tesfa, T. , & Getu, S. (2020). Estimation of technical efficiency of black cumin (*Nigella sativa* L.) farming in northwest Ethiopia: A stochastic frontier approach. Journal of Economic Structures, 9(1), 1–14.

[fsn33982-bib-0029] Dey, P. , Kundu, A. , Kumar, A. , Gupta, M. , Lee, B. M. , Bhakta, T. , Dash, S. , & Kim, H. S. (2020). Analysis of alkaloids (indole alkaloids, isoquinoline alkaloids, tropane alkaloids). In Recent advances in natural products analysis (pp. 505–567). Elsevier.

[fsn33982-bib-0030] Fawzy, Z. F. , El‐Shal, Z. S. , Li, Y. , Zhu, O. , & Sawan, O. M. (2012). Response of garlic (*Allium sativum* L.) plants to foliar spraying of some bio‐stimulants under sandy soil condition. Journal of Applied Sciences Research, 6, 770–776.

[fsn33982-bib-0031] Ghafoor, A. , & Ahmad, Z. (2003). Exploitation of *Vigna mungo* (L.) Hepper germplasm using multivariate analysis based on agronomic traits. Pakistan Journal of Botany, 35(2), 187–196.

[fsn33982-bib-0032] Gholamnezhad, Z. , Havakhah, S. , & Boskabady, M. H. (2016). Preclinical and clinical effects of *Nigella sativa* and its constituent, thymoquinone: A review. Journal of Ethnopharmacology, 190, 372–386. 27364039 10.1016/j.jep.2016.06.061

[fsn33982-bib-0033] Giridhar, K. , Reddy, G. , Surya Kumari, S. , & Naram Naidu, L. (2015). Nigella: A seed spice of blessing. Spice India.

[fsn33982-bib-0034] Hallajzadeh, J. , Milajerdi, A. , Mobini, M. , Amirani, E. , Azizi, S. , Nikkhah, E. , Bahadori, B. , Sheikhsoleimani, R. , & Mirhashemi, S. M. (2020). Effects of *Nigella sativa* on glycemic control, lipid profiles, and biomarkers of inflammatory and oxidative stress: A systematic review and meta‐analysis of randomized controlled clinical trials. Phytotherapy Research, 34(10), 2586–2608. 32394508 10.1002/ptr.6708

[fsn33982-bib-0035] Haque, M. , Singh, R. , Nadeem, A. , Rasool, S. , Wani, J. A. , Khan, A. , Ashafaq, M. , Makeen, H. A. , & Zehra, U. (2022). *Nigella sativa*: A promise for industrial and agricultural economic growth. In Black Seeds (Nigella Sativa) (pp. 439–460). Elsevier.

[fsn33982-bib-0036] Hassan, F. A. S. , & Ali, E. F. (2013). A comparative study between traditional mineral nutrition and alternative sources on anise plant. European Journal of Scientific Research, 106(2), 201–212.

[fsn33982-bib-0037] Herrera Paredes, S. , & Lebeis, S. L. (2016). Giving back to the community: Microbial mechanisms of plant–soil interactions. Functional Ecology, 30(7), 1043–1052.

[fsn33982-bib-0038] Ishtiaq, S. , Ashraf, M. , Hayat, M. Q. , & Asrar, M. (2013). Phytochemical analysis of *Nigella sativa* and its antibacterial activity against clinical isolates identified by ribotyping. International Journal of Agriculture and Biology, 15(6), 1560–1573.

[fsn33982-bib-0039] Jang, J. , Hur, H. G. , Sadowsky, M. J. , Byappanahalli, M. N. , Yan, T. , & Ishii, S. (2017). Environmental Escherichia coli: Ecology and public health implications—A review. Journal of Applied Microbiology, 123(3), 570–581. 28383815 10.1111/jam.13468

[fsn33982-bib-0040] Javed, S. , Shahid, A. A. , Haider, M. S. , Umeera, A. , Ahmad, R. , & Mushtaq, S. (2012). Nutritional, phytochemical potential and pharmacological evaluation of *Nigella Sativa* (Kalonji) and *Trachyspermum Ammi* (Ajwain). Journal of Medicinal Plant Research, 6(5), 768–775.

[fsn33982-bib-0041] Javed, S. , Shoaib, A. , Mahmood, Z. , Mushtaq, S. , & Iftikhar, S. (2012). Analysis of phytochemical constituents of *Eucalyptus citriodora* L. responsible for antifungal activity against post‐harvest fungi. Natural Product Research, 26(18), 1732–1736. 21999598 10.1080/14786419.2011.607451

[fsn33982-bib-0042] Kabir, Y. , Shirakawa, H. , & Komai, M. (2019). Nutritional composition of the indigenous cultivar of black cumin seeds from Bangladesh. Progress in Nutrition, 21, 428–434.

[fsn33982-bib-0043] Kamal, A. (2014). Phytochemical screening of *Syzygium cumini* seeds. Indian Journal of Plant Sciences, 3(4), 1–4.

[fsn33982-bib-0044] Kaushik, N. , & Barmanray, A. (2022). A study on physico‐chemical properties and nutritional profile of an indigenous cultivar‐black cumin (*Nigella sativa* L.). International Journal of Food and Nutritional Sciences, 7, 379–391.

[fsn33982-bib-0045] Kazemi, M. (2014). Phytochemical composition, antioxidant, anti‐inflammatory and antimicrobial activity of *Nigella sativa* L. essential oil. Journal of Essential Oil Bearing Plants, 17(5), 1002–1011.

[fsn33982-bib-0046] Kheiry, A. , Arghavani, M. , & Khastoo, M. (2016). Effects of organic fertilizers application on morphophysiological characteristics of calendula (*Calendula officinalis* L.). Iranian Journal of Medicinal and Aromatic Plants Research, 31(6), 1047–1057.

[fsn33982-bib-0047] Kıralan, M. (2014). Changes in volatile compounds of black cumin (*Nigella sativa* L.) seed oil during thermal oxidation. International Journal of Food Properties, 17(7), 1482–1489.

[fsn33982-bib-0048] Kumar, S. , Sindhu, S. S. , & Kumar, R. (2022). Biofertilizers: An ecofriendly technology for nutrient recycling and environmental sustainability. Current Research in Microbial Sciences, 3, 100094. 35024641 10.1016/j.crmicr.2021.100094 PMC8724949

[fsn33982-bib-0049] Madhukar, C. (2013). Phytochemical screening of cumin seeds extract. Rep Opinion, 5(1), 57–58.

[fsn33982-bib-0050] Majeed, A. , Muhammad, Z. , Ahmad, H. , Hayat, S. S. S. , Inayat, N. , & Siyyar, S. (2021). *Nigella sativa* L.: Uses in traditional and contemporary medicines–an overview. Acta Ecologica Sinica, 41(4), 253–258.

[fsn33982-bib-0051] Mamun, M. A. , & Absar, N. (2018). Major nutritional compositions of black cumin seeds–cultivated in Bangladesh and the physicochemical characteristics of its oil. International Food Research Journal, 25(6), 2634–2639.

[fsn33982-bib-0052] Mariod, A. A. , Ibrahim, R. M. , Ismail, M. , & Ismail, N. (2009). Antioxidant activity and phenolic content of phenolic rich fractions obtained from black cumin (*Nigella sativa*) seedcake. Food Chemistry, 116(1), 306–312. 10.1016/j.foodchem.2009.02.051

[fsn33982-bib-0053] Nadeem, M. , Tehreem, S. , Ranjha, M. M. A. N. , Ahmad, A. , Din, A. , Din, G. M. U. , Javeria, S. , Riaz, M. N. , & Siddeeg, A. (2022). Probing of ultrasonic assisted pasteurization (UAP) effects on physicochemical profile and storage stability of jambul (*Syzygium cumini* L.) squash. International Journal of Food Properties, 25(1), 661–672.

[fsn33982-bib-0054] Naderifar, M. , & Daneshian, J. (2012). Effect of seed inoculation with *Azotobacter* and *Azospirillum* and different nitrogen levels on yield and yield components of canola (*Brassica napus* L.). Iranian Journal of Plant Physiology, 3(1), 619–626.

[fsn33982-bib-0055] Naz, H. (2011). *Nigella sativa*: The miraculous herb. Pakistan Journal of Biochemistry and Molecular Biology, 44(1), 44–48.

[fsn33982-bib-0056] Olad, A. , Zebhi, H. , Salari, D. , Mirmohseni, A. , & Tabar, A. R. (2018). Slow‐release NPK fertilizer encapsulated by carboxymethyl cellulose‐based nanocomposite with the function of water retention in soil. Materials Science and Engineering: C, 90, 333–340. 29853099 10.1016/j.msec.2018.04.083

[fsn33982-bib-0057] Orhan, E. , Esitken, A. , Ercisli, S. , Turan, M. , & Sahin, F. (2006). Effects of plant growth promoting rhizobacteria (PGPR) on yield, growth and nutrient contents in organically growing raspberry. Scientia Horticulturae, 111(1), 38–43.

[fsn33982-bib-0058] Paarakh, P. M. (2010). *Nigella sativa* Linn.–A comprehensive review. Indian Journal of Natural Products and Resources, 1(4), 409–429.

[fsn33982-bib-0059] Pagnani, G. , Pellegrini, M. , Galieni, A. , D'Egidio, S. , Matteucci, F. , Ricci, A. , Stagnari, F. , Sergi, M. , Sterzo, C. L. , & Pisante, M. (2018). Plant growth‐promoting rhizobacteria (PGPR) in *Cannabis sativa* ‘Finola’ cultivation: An alternative fertilization strategy to improve plant growth and quality characteristics. Industrial Crops and Products, 123, 75–83.

[fsn33982-bib-0060] Pavankumar, D. S. , Bn, M. P. , Umesha, K. , Shivanna, M. , Shankarappa, T. H. , & Halesh, G. K. (2018). Influence of plant growth promoting rhizobacteria and plant growth regulators on growth and yield of black cumin (*Nigella sativa* L.) VAR. NS‐44. Journal of Pharmacognosy and Phytochemistry, 7(3S), 1–4.

[fsn33982-bib-0061] Pavithra, K. , & Vadivukkarasi, S. (2015). Evaluation of free radical scavenging activity of various extracts of leaves from *Kedrostis foetidissima* (Jacq.) Cogn. Food Science and Human Wellness, 4(1), 42–46.

[fsn33982-bib-0062] Pedan, V. , Fischer, N. , & Rohn, S. (2016). An online NP‐HPLC‐DPPH method for the determination of the antioxidant activity of condensed polyphenols in cocoa. Food Research International, 89, 890–900.

[fsn33982-bib-0063] Pii, Y. , Mimmo, T. , Tomasi, N. , Terzano, R. , Cesco, S. , & Crecchio, C. (2015). Microbial interactions in the rhizosphere: Beneficial influences of plant growth‐promoting rhizobacteria on nutrient acquisition process. A review. Biology and Fertility of Soils, 51, 403–415.

[fsn33982-bib-0064] Priyom, S. , Islam, M. , Islam, M. , & Shumi, W. (2022). *Microbial technology—a sustainable alternative to improve concrete quality*. Paper presented at the Advances in Civil Engineering: Select Proceedings of ICACE 2020.

[fsn33982-bib-0065] Rabbani, M. A. , Ghafoor, A. , & Masood, M. S. (2011). NARC‐kalonji: An early maturing and high yielding variety of *Nigella sativa* released for cultivation in Pakistan. Pakistan Journal of Botany, 43, 191–195.

[fsn33982-bib-0066] Reddy, S. H. , Al‐Kalbani, A. S. , & Al‐Rawahi, A. S. (2018). Studies on phytochemical screening‐GC‐MS characterization, antimicrobial and antioxidant assay of black cumin seeds (*Nigella sativa*) and senna alexandria (*Cassia angustifolia*) solvent extracts. International Journal of Pharmaceutical Sciences and Research, 9(2), 490–497.

[fsn33982-bib-0067] Saleh, F. A. , El‐Darra, N. , Raafat, K. , & El Ghazzawi, I. (2018). Phytochemical analysis of *Nigella sativa* L. utilizing GC‐MS exploring its antimicrobial effects against multidrug‐resistant bacteria. Pharmacognosy Journal, 10(1), 99–105.

[fsn33982-bib-0068] Shafodino, F. S. , Lusilao, J. M. , & Mwapagha, L. M. (2022). Phytochemical characterization and antimicrobial activity of *Nigella sativa* seeds. PLoS One, 17(8), e0272457. 35926002 10.1371/journal.pone.0272457 PMC9352024

[fsn33982-bib-0069] Singh, B. , & Kaur, A. (2018). Control of insect pests in crop plants and stored food grains using plant saponins: A review. LWT, 87, 93–101.

[fsn33982-bib-0070] Sylvie, D. D. , Anatole, P. C. , Cabral, B. P. , & Veronique, P. B. (2014). Comparison of in vitro antioxidant properties of extracts from three plants used for medical purpose in Cameroon: *Acalypha racemosa, Garcinia lucida* and *Hymenocardia lyrata* . Asian Pacific Journal of Tropical Biomedicine, 4, S625–S632.

[fsn33982-bib-0071] Teshome, W. , & Anshiso, D. (2019). Assessment of production and utilization of black cumin (*Nigella sativa*) at the Oromia regional state, Ethiopia. Asian Journal of Agricultural Extension, Economics & Sociology, 31(3), 1–12.

[fsn33982-bib-0072] Tian, W. , Chen, G. , Zhang, G. , Wang, D. , Tilley, M. , & Li, Y. (2021). Rapid determination of total phenolic content of whole wheat flour using near‐infrared spectroscopy and chemometrics. Food Chemistry, 344, 128633. 33223296 10.1016/j.foodchem.2020.128633

[fsn33982-bib-0073] Tinna, D. , Garg, N. , Sharma, S. , Pandove, G. , & Chawla, N. (2020). Utilization of plant growth promoting rhizobacteria as root dipping of seedlings for improving bulb yield and curtailing mineral fertilizer use in onion under field conditions. Scientia Horticulturae, 270, 109432.

[fsn33982-bib-0074] Tong, Z. , He, W. , Fan, X. , & Guo, A. (2022). Biological function of plant tannin and its application in animal health. Frontiers in Veterinary Science, 8, 803657. 35083309 10.3389/fvets.2021.803657 PMC8784788

[fsn33982-bib-0075] Tura, A. M. , Debisa, M. D. , Tulu, E. D. , & Tilinti, B. Z. (2023). Evaluation of proximate, phytochemical, and heavy metal content in black cumin and fenugreek cultivated in Gamo zone, Ethiopia. International Journal of Food Science, 2023, 3404674. 36684412 10.1155/2023/3404674 PMC9859698

[fsn33982-bib-0076] Uttara, B. , Singh, A. V. , Zamboni, P. , & Mahajan, R. T. (2009). Oxidative stress and neurodegenerative diseases: A review of upstream and downstream antioxidant therapeutic options. Current Neuropharmacology, 7(1), 65–74. 19721819 10.2174/157015909787602823 PMC2724665

[fsn33982-bib-0077] Valadabadi, S. A. , & Farahani, H. A. (2011). Investigation of biofertilizers influence on quantity and quality characteristics in *Nigella sativa* L. Journal of Horticulture and Forestry, 3(3), 88–92.

[fsn33982-bib-0078] Wang, Y. , Zhang, Y. , Liu, X. , Wang, J. , Wei, L. , & Feng, Y. (2014). Effect of a hydrophilic head group on krafft temperature, surface activities and rheological behaviors of erucyl amidobetaines. Journal of Surfactants and Detergents, 17(2), 295–301.

[fsn33982-bib-0079] Yessuf, A. M. (2015). Phytochemical extraction and screening of bio active compounds from black cumin (*Nigella sativa*) seeds extract. American Journal of Life Sciences, 3(5), 358–364.

